# Universal opt-in HIV, HBV and HCV testing in an emergency department: implementation and outcomes of a comprehensive screening program

**DOI:** 10.1007/s15010-025-02710-w

**Published:** 2025-12-15

**Authors:** Kira Sophia Hülsdünker, David Grieser, Pascal Migaud, Daniela Drauz, Keikawus Arastéh, Hartmut Stocker

**Affiliations:** 1 Department of Infectious Diseases, St. Joseph Krankenhaus Berlin - Tempelhof, Wüsthoffstrasse 15, 12101 Berlin, Germany; 2 Charité - Universitätsmedizin Berlin, Charitéplatz 1, 10117 Berlin, Germany

**Keywords:** Blood borne virus infections (BBV), Human Immunodeficiency Virus (HIV), Hepatitis B Virus (HBV), Hepatitis C Virus (HCV), Opt-out testing, Opt-in testing, Emergency department (ED)

## Abstract

**Purpose:**

To evaluate a Blood Born Virus (BBV) infection screening program in an emergency department (ED) located in an urban setting with an intermediate prevalence of undiagnosed BBV infections.

**Methods:**

The program in the ED of the St. Joseph Hospital, Berlin, Germany, was active from June 2021 through April 2024. Patients aged 18–68 undergoing routine blood sampling were eligible for opt-in screening. We analyzed testing uptake, temporal trends, positivity rates, and linkage to care.

**Results:**

A total of 23,118 cases were eligible for testing. Screening was offered to 2670 cases (11.5%). 2440 (91.4%) consented of whom 2406 were tested. Testing volumes remained below 11% of the eligible population.

Among 2406 cases, 78 (3.2%) individuals were found to have at least one BBV infection.

HIV infection was detected in 36 (1.5%) individuals. 12 individuals (0.5%) had previously undiagnosed HIV infection (median [range] CD4 count: 213/µL [66–794]). Linkage to care was successful in 50.0%.

HBV was found in 16 (0.7%) individuals, with 6 (0.2%) previously undiagnosed individuals; linkage to care was achieved in 33.3%.

HCV was confirmed in 38 (1.6%) individuals, including 13 (0.5%) previously undiagnosed individuals; linkage to care was achieved in 15.4%.

Homelessness, substance use, and lack of health insurance coverage were key barriers to successful linkage.

**Conclusions:**

Universal BBV testing in an urban ED proved effective in identifying previously undiagnosed infections. However, due to its opt-in design, the program operated below its potential capacity. Linkage to care was often unsuccessful, largely due to structural barriers.

## Introduction

Blood-borne viruses (BBVs), including Human Immunodeficiency Virus (HIV), Hepatitis B Virus (HBV) and Hepatitis C Virus (HCV) are important pathogens with major implications for individual and public health. Despite breakthroughs in BBV research and treatment, they continue to be a global public health challenge [1], with 304 million people living with HBV and HCV (2022) [2] and 40.8 million people living with HIV (2024) worldwide [3]. In particular, late diagnosis remains a critical barrier to achieving optimal health outcomes and preventing transmission [4]. Early diagnosis through effective testing strategies is essential, as timely initiation of therapy improves individual health outcomes and reduces transmission risk by suppressing viral replication (HIV, HBV) or achieving cure (HCV) [5, 6]. The UNAIDS 95-95-95 targets aim for 95% of people living with HIV to know about their diagnosis, 95% of those diagnosed to receive antiretroviral therapy, and 95% of those on treatment to achieve viral suppression until 2030. For viral hepatitis, the World Health Organization’s (WHO’s) Global Health Sector Strategy aims to reduce global incidence by 90% and mortality by 65% until 2030, compared to 2015. These objectives serve not only to promote individual health, but also to create incentives for overcoming structural barriers in access to diagnostics and treatment [7]. The following sections illustrate recent developments in testing strategies, exemplified through the case of HIV.

Historically, provider initiated HIV testing was primarily risk-based and targeted individuals with specific risk factors or certain clinical indicators [8]. This approach proved insufficient in identifying cases during the asymptomatic phase. Several studies demonstrated that risk-based screening missed a significant part of HIV infections in healthcare settings [9]. Recognition of the limitations of targeted testing led to the development of more extensive approaches. In 2006, the CDC fundamentally changed its philosophy by recommending routine HIV screening in healthcare settings. This new guidance recommended testing all patients between 13 and 64 years of age [8]—an approach that moved away from traditional risk-based testing, after cost-effectiveness and improved case detection rates through routine screening had been demonstrated [10]. This recommendation is for the US, where the prevalence of undiagnosed HIV infection is higher than in Germany, making it an unsuitable strategy for settings with lower prevalences. However, implementing testing in emergency departments (EDs) that serve urban populations may increase the pretest probability, even in areas with lower prevalences, thereby supporting the rationale of this strategy. Emergency departments could play a vital role in BBV testing, particularly for populations with limited access to regular healthcare as well as for some migrant populations who tend to attend hospitals for primary care. Studies have demonstrated feasibility and effectiveness of universal testing in ED settings, if implemented sensibly [9, 11].

In Germany, the overall prevalence of HIV is estimated to be around 0,1%. The Robert Koch Institut (RKI), Germany’s national public health agency, estimated the number of annual new infections to be 1900 in 2022 and 2200 in 2023. Approximately 33% of new diagnoses were classified as late diagnoses with advanced immunodeficiency, with half of these cases already presenting with AIDS-defining diseases. It is important to note that the RKI defines late HIV diagnosis as a diagnosis made either with a CD4 cell count of < 200 cells/µL or with an AIDS-defining condition, which differs from the internationally accepted definition of < 350 cells/µL or AIDS. As a result, the proportion of people identified as having been diagnosed late is lower compared to settings that apply the broader definition. The proportion of undiagnosed infections is currently estimated to be 8%, not yet including undiagnosed infections among people born outside of Germany, who acquired their infection abroad [12].

With an HIV prevalence of 417 per 100,000 individuals (0.4%), Berlin stands out as having one of the highest rates in Germany, highlighting its unique epidemiological profile [13]. More than 1040 people with unknown HIV infection were estimated to live in Berlin in 2023. 300 people were newly diagnosed in that same year with approximately a third of them in advanced disease stages (CD4 cell count < 200/µl and / or AIDS-defining conditions) [14]. Groups likely at higher risk of late diagnosis are heterosexual men and women, people with a background of migration and people of age [12, 15].

Implementing a universal testing strategy in a high-prevalence setting such as Berlin could lead to earlier diagnoses, reduced transmission, and lower healthcare costs [16]. This study retrospectively evaluates a provider-initiated, universal, opt-in HIV, HBV and HCV testing project in an emergency department in central Berlin.

## Methods

### Study design and setting

This retrospective observational study analyzed data from a universal BBV screening program for HIV, HBV and HCV. The program was implemented at the St. Joseph Hospital in Berlin, Germany, a 506-bed tertiary care hospital serving an urban population of approximately 400,000 residents. The hospital's emergency department manages approximately 50,000 patient visits annually [17]. The study period spanned from 1 June 2021 to 29 April 2024. Because of interruptions in February and April 2022, and between October 2023 and January 2024 only 29 non-continuous months within this 35-month period were included in the analysis.

### Eligibility for BBV infection screening

The program aimed to implement universal BBV screening using an opt-in approach among emergency department patients meeting predefined eligibility criteria. Inclusion criteria were age between 18 and 68 years *and* the requirement for blood sample collection as part of routine clinical care.

Patients fulfilling these criteria were asked to provide informed consent to participate in the screening program. Individuals unable or unwilling to provide consent and those presenting due to occupational injuries were excluded.

### Testing protocol and patient education

A comprehensive patient education and consent process was implemented. Information about the screening program was disseminated through multiple channels, including informational posters and flyers placed in waiting areas and triage rooms. Nursing staff, who received training in patient education and counseling before the project start, provided information about the screening program prior to blood collection. Patients received the information about the testing offer in a condensed format on a postcard, which simultaneously functioned as the consent form. These cards were available in German, English, Arabic and Turkish. During the first 17 months of the program, consent forms were also distributed to patients who declined participation. Cards collected from individuals who provided and those who declined consent served to estimate program acceptance rates among eligible patients for the entire study period.

### Laboratory testing and result management

The screening protocol comprised three distinct tests: a fourth-generation HIV antigen/antibody combination assay, HBsAg testing for HBV, and anti-HCV antibody testing for HCV. Blood collection was integrated into routine clinical care, requiring only one additional collection tube. Test results were available within a maximum of three hours after sample collection. All positive screening results underwent confirmation according to standard laboratory algorithms. Test results were communicated to patients by the physicians in charge. Patients with positive results received immediate referral options to the hospital's infectious disease department. Patients who left the emergency department before positive test results arrived were contacted via phone or mail. Patients were counseled regarding available treatment options. They were either provided with a direct appointment with an outpatient infectious disease specialist or received a list of specialists located near their place of residence. In cases where no health insurance coverage was in place, patients were referred to the 'Clearingstelle'—an institution that assesses the possibility of obtaining health insurance or, alternatively, covers the cost of outpatient care.

### Data collection and management

A data collection protocol was established to systematically gather participant information. Demographic data included age, gender, test date, and results for all three tests. For patients with positive test results that represented previously undiagnosed infections, additional information was retrospectively extracted from the hospital information system. This included diagnostic classification (known vs. previously undiagnosed infection, or false-positive result), care status for known infections (actively linked to care vs. lost to follow-up), and presenting complaints. For patients with previously undiagnosed infections, presenting complaints, infection-related symptoms, CD4 cell counts and viral loads (if applicable), as well as information on linkage to care, were extracted from the hospital information system. Substance use as well as social determinants were also documented, including country of origin, health insurance coverage, and housing status.

Additionally, we extracted the total number of patients presenting to the ED during the study period from the hospital information system to calculate the testing rate. Patients with multiple visits on the same day shared one case number and were counted only once. The demographic data (sex and age) of the tested population were compared with those of the entire patient cohort.

To ensure data security and privacy, all collected information was stored in a password-protected database within the hospital's secure network. Personal identifiers were replaced with study-specific identification numbers in the analysis dataset. Regular monitoring and validation checks were performed to maintain data quality and integrity.

### Statistical analysis

All statistical analyses were performed using R version 4.4.2 (R Foundation for Statistical Computing, Vienna, Austria) [18]. Descriptive statistics were calculated for all variables, with categorical data summarized as frequencies and percentages, and continuous variables presented as medians with ranges. All results were rounded to one decimal place.

Primary endpoints were testing coverage—defined as the proportion of eligible patients tested and the test acceptance rate—and diagnostic yield, measured by overall positivity and rates of previously undiagnosed infections.

Secondary endpoints included demographic characteristics and clinical presentations, with linkage to care examined separately. Demographic analyses included age and sex, as well as housing status, substance use and insurance coverage. Age and sex distributions of tested individuals and those eligible, but not tested were compared, using a Wilcoxon rank-sum and a Chi-squared test. Three observations were excluded from the Chi-squared test due to low expected cell frequencies (“diverse” gender category). Clinical presentation analyses examined symptoms at presentation, CD4 cell count, and viral loads among patients with previously undiagnosed HIV infection. Finally, data on linkage to care were analyzed to assess follow-up and integration into treatment services.

Patients with multiple ED visits on the same day were counted only once. However, repeated visits on different days could not be reliably identified. As a result, the reported number of cases likely exceeds the actual number of unique individuals.

Missing values for individual variables were reported where applicable. Unless otherwise stated, all variables had complete data. In cases where missing values occurred, percentages were calculated based on the total number of observations. Continuous variables like CD4 cell count or viral load were summarized using available data only; observations with missing values were omitted from the analysis of the respective variable.

## Results

### Overall testing program outcomes

**Fig. 1:**
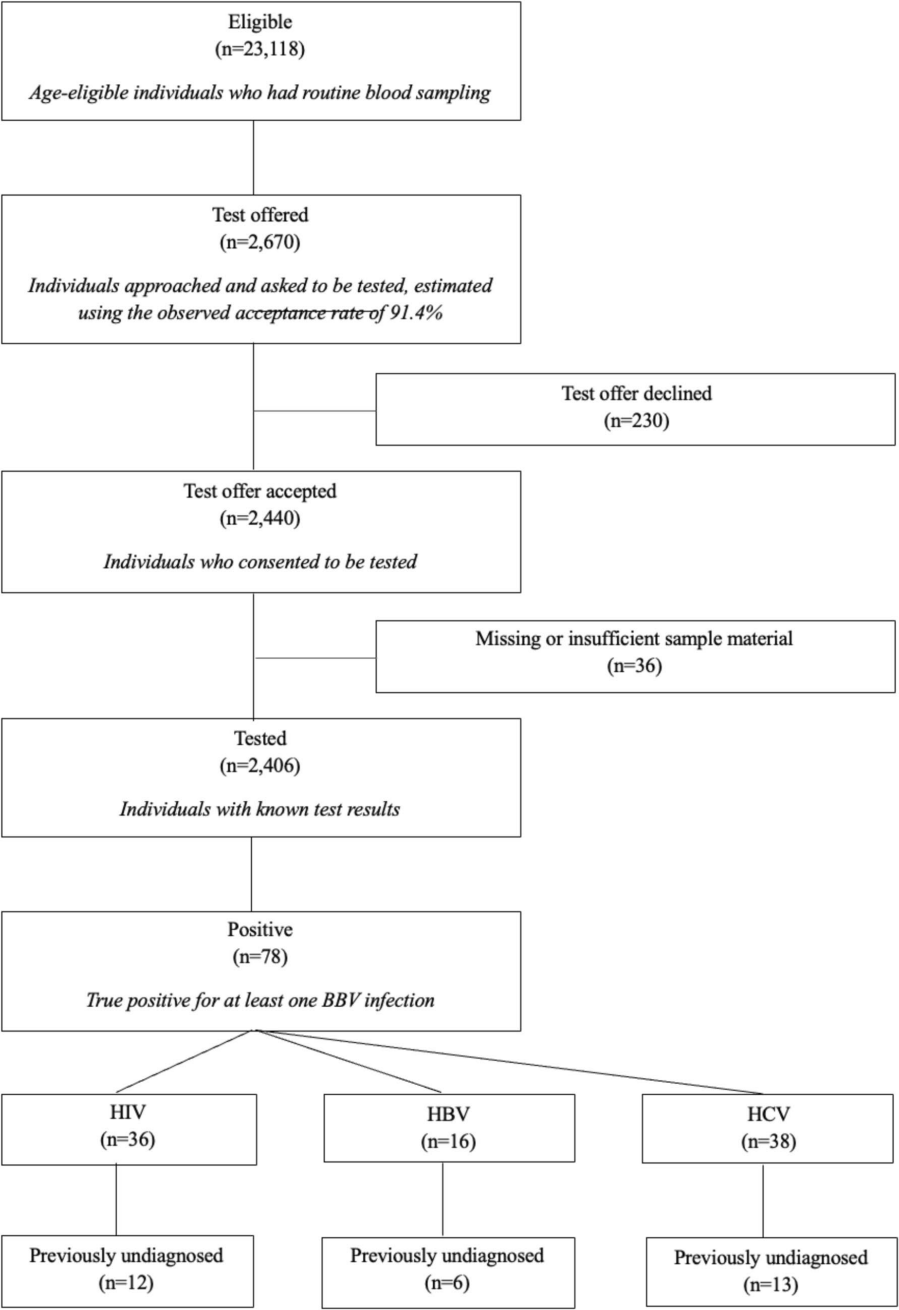
Subject disposition chart

During the study period, 35,584 cases met the eligibility criteria of an age between 18 and 68. 23,118 (65.0%) had a routine blood sample collection and of those, 2440 (10.6%) were both offered a test and consented to participate in the screening program. Testing was completed in 2406 cases; in a few instances, analysis was precluded by missing or insufficient sample material (Fig. 1).

During the initial 17 months, when consent documentation included both acceptances and declinations, the test acceptance rate among those approached was 91.4%. Based on this acceptance rate, we estimated the number of individuals who were offered testing during the entire study period and arrived at an estimated total of 2670. This results in an approach rate of 11.5%.

To assess potential selection bias, tested and untested individuals were compared regarding their demographic characteristics. The median [range] age was 37 [18–68] years among tested and 39 [18–68] years among untested individuals, a difference that was statistically significant (Wilcoxon rank-sum test, *p* < 0.001). The proportion of women was 51.3% and 60.2% in the two groups, respectively, and this difference was also statistically significant (χ^2^ test, *p* < 0.001) (Table 1).

**Table 1:** Age and sex distribution of eligible, untested, tested, and individuals who tested true positive (including both known and previously undiagnosed infections)

	Eligible	Untested	Tested	HIV (+)	HBV (+)	HCV (+)
	n = 23,118	n = 20,712	n = 2,406	n = 36	n = 16	n = 38
Age [years],						
median [range]	39 [18–68]	39 [18–68]*	37 [18–68]*	45.5 [28–65]	47 [27–66]	38 [21–66]
Sex [n] (%)						
Female	13,695 (59.2)	12,460 (60.2)^#^	1,235 (51.3)^#^	2 (5.6)	7 (43.8)	10 (26.3)
Male	9,420 (40.7)	8,249 (39.8)^#^	1,171 (48.7)^#^	34 (94.4)	9 (56.3)	28 (73.7)
Diverse	3 (0.0)	3 (0.0)	0 (0.0)	0 (0.0)	0 (0.0)	0 (0.0)

*Age differed significantly between tested and untested individuals (Wilcoxon rank-sum test, *p* < 0.001)

^#^Sex distribution differed significantly between tested and untested individuals (χ ^2^ test, *p* < 0.001)

Among the 2406 cases tested, 78 (3.2%) had true positive screening results for at least one of the BBV infections. Specifically, 36 individuals (1.5%) tested true positive for HIV, 16 (0.7%) for HBV, and 38 (1.6%) for HCV. 2 individuals (0.1%) tested positive for both HIV and HBV, 9 (0.4%) for both HIV and HCV and one for both HBV and HCV (Table 1).

### HIV screening outcomes

Among the 2406 cases tested, 36 had confirmed positive HIV screening results. 12 of those (33.3%) represented new diagnoses, corresponding to a rate of 0.5% previously undiagnosed HIV infections among tested patients. One patient had a confirmed positive HIV test, but the remaining data were missing. Among the 23 patients with known HIV infection, 20 (87.0%) had an existing link to care, while 3 (13.0%) were identified as lost to follow-up. Of those lost to follow-up, 2 could not be re-linked to care, for one patient information was missing.

8 of the 12 individuals with previously undiagnosed HIV infection had a CD4 cell count documented with a median [range] of 213 cells/µL [66–794]. 6/8 had CD4 cell counts below 350 cells/µL, indicating an advanced disease stage. The median [range] viral load at diagnosis was 5.7 copies log_₁₀_/mL [4.2–6.8] among the 9 individuals whose viral loads were measured. Among the 12 patients with previously undiagnosed infection, one presented with an AIDS-defining condition (*Pneumocystis* pneumonia). The presenting complaints among newly diagnosed patients are listed in Table 2.

**Table 2:** Patient characteristics, presenting complaints, CD4 cell counts and HIV RNA in individuals with previously undiagnosed BBV infection

Individual	Previously undiagnosed BBV infection			Presenting complaint	Sex	Age [years]	CD4 count [cells/µL]	HIV RNA [copies log₁₀ /mL]
	HIV	HBV	HCV					
1	(+)			Bolus sensation	M	41	223	n.d
2	(+)			Anemia	M	50	475	6.1
3	(+)			Kidney failure	M	37	139	4.2
4	(+)			Fear of infection	F	28	n.d	n.d
5	(+)			Skin infection	M	31	165	6.4
6	(+)			Anal pain	M	36	203	5.7
7	(+)			Pneumonia	M	53	66	5.7
8	(+)			Dysphagia	M	32	265	6.8
9	(+)			Diarrhea	M	34	794	5.5
10	(+)		(+)	Skin infection	M	30	n.d	4.6
11	(+)		(+)	Intoxication	M	41	n.d	n.d
12	(+)		(+)	Intoxication	M	39	n.d	5.0
13		(+)		Chest pain	M	27	n.a	n.a
14		(+)		Abdominal pain	F	30	n.a	n.a
15		(+)		Abdominal pain	M	52	n.a	n.a
16		(+)		Rectal bleeding	M	48	n.a	n.a
17		(+)		Anemia	F	46	n.a	n.a
18		(+)		Abdominal pain	F	31	n.a	n.a
19			(+)	Skin infection	M	21	n.a	n.a
20			(+)	Skin infection	M	26	n.a	n.a
21			(+)	Skin infection	M	32	n.a	n.a
22			(+)	Abdominal pain	M	53	n.a	n.a
23			(+)	Skin infection	M	42	n.a	n.a
24			(+)	Intoxication	F	22	n.a	n.a
25			(+)	Skin infection	F	35	n.a	n.a
26			(+)	Skin infection	F	49	n.a	n.a
27			(+)	Pneumonia	M	34	n.a	n.a
28			(+)	Sepsis	M	25	n.a	n.a
	**HIV**	**HBV**	**HCV**		F [%]	median [range]	median [range]	median [range]
	(±)	(±)	(±)		21.4	35 [21—53]	n.a	n.a
	(+)				8.3	36.5 [28—53]	213 [66—794]	5.7 [4.2—6.8]
		(+)			50.0	38.5 [27—52]	n.a	n.a
			(+)		23.1	34 [21—53]	n.a	n.a

*F* Female, *M* Male, *(+)* positive test result, *n.a.* not applicable, *n.d.* not determined, *(±)* this line summarizes the patient characteristics of patients with ≥ 1 positive BBV test result

Individuals with previously undiagnosed HIV infection had a median [range] age of 36.5 [28–53] years. Among these, 11 (91.7%) were male and 1 (8.3%) was female (Table 2). Regarding country of origin, 5 (41.7%) were born in Germany, 6 (50.0%) outside of Germany, and for 1 (8.3%) individual this information was unavailable. Homelessness was reported by 5 (41.7%) individuals, and 4 (33.3%) lacked health insurance. In terms of transmission risk, 5 (41.7%) identified as men who have sex with men (MSM), and 4 (33.3%) reported regular intravenous drug use. Successful linkage to care was achieved for 6 (50.0%) of the patients with previously undiagnosed infection, while another 5 (41.7%) could not be successfully linked. For 1 patient information was missing as she left Germany to seek care in her home country. 1/6 patients, who could successfully be linked to care, was homeless, reported intravenous drug use and lacked health insurance coverage. Among patients who could not be linked to care, 4/5 were homeless, 3/5 reported intravenous drug use, and 2/5 lacked health insurance coverage. In total, 50.0% of previously undiagnosed individuals exhibited at least one of the aforementioned vulnerabilities.

### Hepatitis B screening outcomes

HBV screening yielded 18 positive results among the tested population. After confirmatory testing, 16 results were confirmed as true positives. 6 (37.5%) of all confirmed cases were previously undiagnosed, corresponding to a rate of 0.2% previously undiagnosed HBV infections among all tested patients.

Demographic analysis of cases with previously undiagnosed infection revealed a median [range] age of 38.5 [27–52] years, with half being female (Table 2). 3/6 newly diagnosed HBV cases were in individuals born outside of Germany, one was born in Germany and for 2 individuals information was missing. Among previously undiagnosed individuals, 4/10 patients had health care insurance coverage, one patient did not have health care insurance coverage, and for one individual this information was missing. Housing instability was reported by none of the newly diagnosed HBV patients, although we do miss information for one patient.

Successful linkage to care was achieved for 2/6 individuals with previously undiagnosed HBV infection, who both had housing and insurance coverage. 3/6 could not be successfully linked and for one patient information was missing.

### Hepatitis C screening outcomes

HCV screening identified 51 positive individuals (2.1%). Confirmatory testing verified 38 true positive individuals. Among these, 13 (34.2%) represented previously undiagnosed individuals, corresponding to a rate of 0.5% previously undiagnosed HCV infections among all tested patients. Among people with known HCV infections, 13 (52.0%) were engaged in care at the time of screening. 7 (28.0%) were not and for 5 information is missing. Among the 7 previously diagnosed patients who were lost to follow-up, one could be successfully re-engaged in care through the screening program.

The demographic profile of individuals with newly diagnosed HCV infections had a median [range] age of 34 years [21–53], with 3 (23.1%) being female (Table 2). 7 (53.8%) of the previously undiagnosed HCV infections were found in patients born in Germany, 3 (23.1%) in patients born outside of Germany and 3 in patients for whom information is missing. 4 (30.8%) individuals lacked health insurance coverage. For 1 person information is missing. 10 (76.9%) of the previously undiagnosed individuals reported homelessness.

Common presenting complaints of individuals with previously undiagnosed HCV infection included skin infections (38.5%) and drug intoxication (23.1%). Successful linkage to care was achieved for 2 (15.4%) of the individuals newly diagnosed, with both having health insurance coverage, but one being homeless. 5 (38.5%) could not be linked to care and for 6 information is missing.

### Temporal distribution of testing volume

Analysis of testing implementation revealed substantial fluctuations throughout the study period. Initial testing volumes peaked at approximately 240 tests per month in June 2021, followed by a marked decline to near-zero testing rates in the first quarter of 2022. Subsequently, two notable peaks were observed: one in May 2022 with approximately 160 tests and another in August 2023 with approximately 165 tests per month. These cyclical variations temporally corresponded with systematic staff reminders and refresher training sessions.

## Discussion

This study summarizes the evaluation of the first and only universal BBV testing program in a German ED setting.

Offering testing for all three BBV infections (HIV, HBV, and HCV) was a deliberate strategy to reduce the stigma associated with HIV-only testing and to increase acceptance. This approach proved effective: our combined testing strategy achieved higher acceptance rates than those reported in previous studies that focused solely on HIV testing in emergency departments, where acceptance typically ranged from 80 to 90% [19–21] or lower [22, 23]. This finding suggests that combined BBV-testing may enhance patient acceptance rates and destigmatize the testing process. Furthermore, the identification of concurrent infections in 12 of 78 patients with at least one positive test result (15.4%) highlights the public health value of comprehensive BBV screening programs.

Another key element of our implementation model was the integration of testing into routine blood sample collection, minimizing additional staff burden and patient inconvenience. This seems to be an important prerequisite, according to a CDC report from 2021, which showed that, despite the revised recommendations of 2006, less than 1% of patients were tested in primary care settings in the US in recent years [24]. A likely reason may be the setting itself as EDs are specifically characterized by high workloads, high patient turnover, and staff shortages. The integration of tests into existing workflows without significant disruption to emergency care, the integration with existing laboratory infrastructure and the development of streamlined protocols for consent procedures and result notification seem therefore to be important considerations [25–27].

Our findings confirm that testing in EDs in urban areas can effectively identify individuals with previously undiagnosed HIV infections. The diagnostic yield of 0.5% aligns with previous studies in the US [28] and is even higher compared to some other European settings [21, 22, 29–31]. It is a key finding with several important implications. First, this rate exceeds commonly accepted cost-effectiveness thresholds for HIV screening in healthcare settings, suggesting that the testing strategy is not only clinically meaningful but may also be economically justified, however, a potential economic gain still warrants further investigation. Second, the prevalence of newly diagnosed HIV infections in our study population lies well above both the overall HIV prevalence in Berlin as reported by the Robert Koch Institute (RKI) and, more notably, the estimated prevalence of undiagnosed HIV infections. While this could indicate an underestimation in surveillance data, a more plausible explanation is that emergency departments serve populations with a higher baseline prevalence and pre-test probability of HIV infection.

CD4 cell counts at diagnosis in our study were lower than we would expect from national surveillance data [12, 14] and comparable initiatives in other countries [32]. We thus must acknowledge that surveillance data is influenced by case definitions, reporting mechanisms, and population denominators which should be considered, to avoid overinterpretation.

Nevertheless, most individuals with previously undiagnosed HIV infection had not yet developed AIDS-defining conditions at the time of diagnosis. This finding suggests that, although many were diagnosed late in the course of infection, they were not diagnosed too late to benefit from timely initiation of antiretroviral therapy.

The diagnostic yield of 0.2% for new HBV and 0.5% for new HCV infections drastically exceeds surveillance-based incidence estimations for Berlin. This supports the hypothesis that inner-city emergency departments serve populations with higher BBV pretest probabilities even more. Consistent with the surveillance data [33], males where diagnosed more often with HBV and HCV respectively. Similar to the situation with HIV, linkage to care was achieved for only a few of the cases with newly diagnosed HBV and HCV infections. To prevent identified individuals from being lost to follow up after diagnosis, future testing programs need to specifically target homelessness and lack of health insurance as key determinants of unsuccessful linkage to care.

Our testing rate remained low, raising the question of why so few patients were offered a test despite repeated staff training interventions. The major barrier was likely the opt-in model, which requires informed consent. Actively seeking the patient’s consent imposes additional demands on the emergency department staff, which, due to high patient volumes or individual circumstances, may not have been met by the nurses. This argument is supported by significant temporal variations in testing volumes, ranging from near-zero to 240 tests per month, with notable peaks coinciding with staff training sessions. These findings suggest that continuous education and reminder systems are crucial for maintaining consistent screening rates. At the same time, it becomes clear that in an opt-in setting even the most intensive staff education and reminder systems will probably not result in a significant increase in testing rates above approximately 10%. The data indicate that, even during periods with the highest testing activities, a large number of opportunities for testing were missed or in other words, the testing program operated far below it’s potential, a finding that is consistent with the analysis of an opt-in screening program in London [34].

Evidence from England strengthens the premise that an opt-out approach can lead to significantly higher testing rates. In a 2011 pilot project, only 14% of eligible patients were offered testing under an opt-in approach in London emergency departments [34]. In contrast, in the same setting four years later, an opt-out strategy that included combined BBV testing resulted in 28% of patients receiving a test [35]. Overall, the implementation of an opt-out HIV screening program in emergency departments of the NHS England has been remarkably successful: More than 80 hospitals are now offering universal opt-out testing [36], thereby bringing down the rates of undiagnosed HIV infection from 5.8% to approximately 4.5% between 2019 and 2023 [37]. This approach demonstrates the effectiveness of opt-out routine testing in reaching populations that might otherwise be missed [38]. In addition, an Italian study demonstrated a significant gain in both life time and quality with opt-out screening compared to indicator-triggered opt-in screening [39], and an epidemic projection model study in the US provides further evidence for the positive effects of opt-out screening in EDs [40]. Moving from an opt-in to an opt-out strategy marks an important paradigm shift: Despite the absence of specific legal regulations governing the implementation of HIV testing, legal concerns and objections from patient organizations continue to impede the adoption of opt-out HIV testing programs in Germany. Given that HIV is now a treatable chronic condition and effective therapy enables a normal life expectancy, reconsidering the current opt-in versus opt-out paradigm could offer an opportunity to enhance early detection.

What if all eligible patients had been tested in a hypothetical opt-out setting in Berlin? It is unlikely that the rate of undiagnosed infections would have remained as strikingly high. The comparative analyses of the tested versus the untested study population with respect to age and sex suggest that testing was not offered at random. Rather, it appears that the nurses offering the tests made a selective choice, likely influenced by implicit or explicit biases. Therefore, while expanding the program to include all eligible patients that do not opt out would likely have identified a greater absolute number of individuals with BBV infections. However, the overall rate of new diagnoses per test would probably have been lower in a truly universal screening program. A randomized trial in U.S. emergency departments demonstrated this elegantly: although targeted screening yielded a nearly 60% higher proportion of previously undiagnosed HCV infections than universal screening, the absolute number of new HCV diagnoses was greater in the universally screened population [41]. In this context, it remains uncertain whether, under such a hypothetical opt-out model, the rate of newly diagnosed infections per test would have exceeded the cost-effectiveness threshold for each individual BBV. Nonetheless, it is important to note that estimate-driven modelling suggests cost-effectiveness for an opt-out testing strategy in high-prevalence areas in Germany. An opt-out strategy was further estimated to improve linkage to care for individuals with both previously known and newly diagnosed infection [42].

The 50.0% rate of successful linkage to care in individuals with previously undiagnosed HIV infection demonstrates the importance of an efficient referral system. The linkage-to-care rates in this project were lower than those reported in other emergency department-based testing programs [9, 21, 28, 30], which may be attributable to differences in access to the health care system between countries. Our findings suggest that lacking health insurance coverage and being homeless are significant barriers to effective linkage to BBV treatment. This reflects a structural shortcoming of the German healthcare system in addressing the needs of marginalized populations. The problem is most pronounced in the context of HCV (15.4% linkage to care rate), however it sadly confirms the findings of the HCV screening program in US EDs [40]. It is followed by HBV (33.3%) and HIV (50.0%). From a public health perspective, the effectiveness of a costly BBV testing strategy is substantially reduced if not integrated into a systems-based approach that guarantees timely and effective linkage to care for individuals diagnosed. Addressing this critical gap may represent an important future role for non-governmental organizations (NGOs) and community-based actors.

The cost-effectiveness implications of our findings merit consideration. While a detailed economic analysis was beyond this study's scope, previous research suggests that universal HIV screening is cost-effective in settings with prevalence rates of previously undiagnosed HIV infections of > 0.1% [43]. Given Berlin's HIV prevalence of 0.4% [13] and our diagnostic yield, our findings support the economic viability of universal screening in this setting [16]. The finding that a high proportion of individuals with previously undiagnosed HIV infections were diagnosed with low CD4 cell counts, yet still without AIDS-defining conditions highlights the potential for substantial healthcare cost savings through earlier intervention and prevention of complications. Moreover, timely diagnosis and initiation of antiretroviral therapy contribute to reduced HIV transmission rates via treatment as prevention, reinforcing the public health value of early detection.

This initiative is timely as Germany falls short of the UNAIDS 95-95-95 targets. While treatment and viral suppression goals have been met, the diagnosis target remains unmet. Our findings highlight the potential of ED-based universal screening to help close this gap—particularly in high-prevalence urban areas—provided systemic barriers related to social factors and health insurance access are addressed.

This study has several limitations. Its single-center design may limit generalizability, particularly to settings with different prevalence rates, different populations and a different philosophy to access to health insurance. Berlin has a comparatively large population of individuals without access to health insurance coverage, driven by factors such as lack of legal residency status, homelessness, and substance use—challenges typical of major urban centers in Germany and elsewhere. These barriers might be less relevant in countries with a more liberal access to health care. Testing was not offered systematically, and implicit staff bias may have influenced who was approached. Selection bias in this context likely means that individuals with a higher pre-test probability were preferentially tested. This, in turn, limits the generalizability of the findings to a universal screening context, as a certain proportion of non-random test offerings must be assumed. As a retrospective analysis of a pilot program, data collection was not standardized, and misclassification cannot be ruled out. Key information, including linkage to care, was manually retrieved and may be incomplete or inconsistent. Caution is particularly warranted when interpreting linkage-to-care rates, as we only recorded initial connections, not long-term retention. Interpretation here is also limited by a substantial proportion of missing data in linkage-to-care outcomes, which cannot be assumed to be missing at random and may introduce bias. The low approach rate remains unexplained and warrants further qualitative investigation.

## Conclusion

Our study shows that universal BBV testing in a Berlin emergency department is feasible, well accepted, and effective in identifying undiagnosed infections, including a 0.5% yield for previously undiagnosed HIV infections. Combining HIV, HBV, and HCV screening likely reduced stigma and improved uptake. However, low offer rates and inconsistent testing volumes highlight the limitations of an opt-in consent strategy and the need to explore opt-out strategies. Linkage to care remains a challenge, particularly for individuals without health insurance coverage, those with substance use, and homeless people. For ED-based testing to have meaningful public health impact, it must be embedded in a systems-based approach that addresses these structural barriers.

## Data Availability

The datasets generated and analysed are not publicly available due to privacy restrictions, but are available from the corresponding author on reasonable request.

## Abbreviations

BBV: Blood-borne virus
ED: Emergency department
HBV: Hepatitis B Virus
HCV: Hepatitis C Virus
HIV: Human Immunodeficiency Virus
RKI: Robert Koch Institut
WHO: World Health Organization
